# Transitions in hookah (Waterpipe) smoking by U.S. sexual minority adults between 2013 and 2015: the population assessment of tobacco and health study wave 1 and wave 2

**DOI:** 10.1186/s12889-021-10389-5

**Published:** 2021-03-05

**Authors:** Mary Rezk-Hanna, Ian W. Holloway, Joy Toyama, Umme Shefa Warda, Lorree Catherine Berteau, Mary-Lynn Brecht, Linda Sarna

**Affiliations:** 1 School of Nursing, University of California, Los Angeles, 700 Tiverton Ave, 4-254 Factor Building, Los Angeles, CA 90095 USA; 2 Department of Social Welfare, Luskin School of Public Affairs, University of California, Los Angeles , Los Angeles, CA USA

**Keywords:** Hookah, Waterpipe, Sexual minority, Tobacco, Vaping

## Abstract

**Background:**

Tobacco smoking using a hookah (i.e., waterpipe) is a global epidemic. While evidence suggests that sexual minorities (SM) have higher odds of hookah use compared to heterosexuals, little is known about their hookah use patterns and transitions. We sought to examine transitions between hookah smoking and use of other tobacco and electronic (e-) products among SM adults aged 18 years of age and older versus their heterosexual counterparts.

**Methods:**

We analyzed nationally representative data of ever and current hookah smokers from Wave 1 (2013–2014; ever use *n* = 1014 SM and *n* = 9462 heterosexuals; current use *n* = 144 SM and *n* = 910 heterosexuals) and Wave 2 (2014–2015; ever use *n* = 901 SM and *n* = 8049 heterosexuals; current use *n* = 117 SM and *n* = 602 heterosexuals) of the Population Assessment of Tobacco and Health Study. Comparisons between groups and gender subgroups within SM identity groups were determined with Rao-Scott chi-square tests and multivariable survey-weighted multinomial logistic regression models were estimated for transition patterns and initiation of electronic product use in Wave 2.

**Results:**

Ever and current hookah smoking among SM adults (ever use Wave 1: 29% and Wave 2: 31%; current use Wave 1: 4% and Wave 2: 3%) was higher than heterosexuals (ever use Wave 1: 16% and Wave 2: 16%; current use Wave 1: 1% and Wave 2: 1%; both *p* < 0.0001). Among SM adults who reported hookah use at Wave 1, 46% quit hookah use at Wave 2; 39% continued hookah use and did not transition to other products while 36% of heterosexual adults quit hookah use at Wave 2 and 36% continued hookah use and did not transition to other products. Compared with heterosexuals, SM adults reported higher use of hookah plus e-products (Wave 2 usage increased by 65 and 83%, respectively).

**Conclusions:**

Compared to heterosexuals, in addition to higher rates of hookah smoking, higher percentages of SM adults transitioned to hookah plus e-product use between 2013 and 2015. Results have implications for stronger efforts to increase awareness of the harmful effects of hookah as well as vaping, specifically tailored among SM communities.

**Supplementary Information:**

The online version contains supplementary material available at 10.1186/s12889-021-10389-5.

## Background

Tobacco smoking using a hookah (i.e., waterpipe) is a global epidemic [1]. Contributing to hookah’s popularity is the unsubstantiated belief that smoke is detoxified as it passes through water, rendering hookah as a safer tobacco alternative [2–4]. Tobacco and alternative tobacco products are disproportionately being used by sexual minority (SM) adults (i.e., lesbian, gay and bisexual individuals) [5–8]. According to Wave 1 Population Assessment of Tobacco and Health (PATH) Study (2013–2014), 39.8% of lesbian/gay adults and 45.7% of bisexual adults reported current tobacco use, compared to 27.3% of heterosexual individuals [9]. Lesbian and bisexual women (18 years of age and older) had higher odds of experimental and regular use of hookah compared to heterosexual women [10]. Similarly, gay identified men > 25 years of age had higher odds of experimental hookah use.

While prevalence rates provide useful information about hookah use among SM adults, to date, virtually nothing is known about how SM hookah smokers have quit or transitioned over time to other tobacco products, including electronic nicotine delivery systems such as e-cigarettes. Understanding changes in tobacco use behavior over time is imperative for providing insight into the net population health impact of tobacco use as well as how to support quit efforts. This is specifically important given the known tobacco-use disparities among SM individuals. Indeed, common smoking risk factors, including stress and depression—experienced at higher rates among SM adults compared to heterosexual adults—have been shown to play a vital role in etiologies of tobacco-related disparities and may make quitting more difficult [11]. Additionally, the tobacco industry has aggressively targeted sexual and racial/ethnic minorities through specifically designed marketing campaigns, community outreach and promotions [12]. Research shows these populations face higher risk of being exposed to online tobacco marketing and are more likely to interact with tobacco-related messages on social media compared to their heterosexual counterparts [13–16]. In particular, SM women have reported more exposure to tobacco industry marketing than heterosexual women [17].

Among the general population, recent longitudinal nationally representative data from PATH study show that while the overall prevalence of tobacco product use decreased (from 28 to 26%) from Wave 1 (2013–2014) to Wave 2 (2014–2015), over half of U.S. adult tobacco users transitioned in product use or combination of products used [18]. Among Wave 1 tobacco users, 72% of young adults (18–24 years of age) transitioned to use other products, including non-combustible and electronic nicotine devices; 20.7% discontinued use completely; and 45.9% of older adults (> 25 years of age) transitioned to other products, with 12.5% discontinuing use completely.

Transitions in hookah use to other tobacco products or quitting all together among SM adults remains unknown. Accordingly, using Wave 1 (2013–2014) and Wave 2 (2014–2015) survey data from the PATH Study, the objective of this study was to characterize transitions between hookah smoking and use of other tobacco products, including cigarettes, cigars, cigarillos, smokeless tobacco, pipe tobacco, snus pouches, dissolvable tobacco and electronic (e-) nicotine products, among SM adults aged 18 years of age and older versus their heterosexual counterparts.

## Methods

### Study design

We used data for adults 18 years and older from Wave 1 (September 12, 2013, to December 14, 2014) and Wave 2 (October 23, 2014, to October 30, 2015) of the PATH Study, a nationally representative, longitudinal cohort study of non-institutionalized adult and youth residents of the U.S. ages 12 and older. The PATH Study was designed to collect data on use patterns, risk perceptions, attitudes and health outcomes associated with tobacco and alternative tobacco products [19]. The PATH study design oversampled adult tobacco users, young adults (aged 18–24) and African-American adults, relative to population proportions. Weighting procedures adjusted for oversampling and allowed for representation of non-institutionalized, civilian US population. A detailed overview of the PATH study design and methods are reported elsewhere [19, 20]. The PATH study was approved by Westat’s Institutional Review Board, and the United States Office of Management and Budget approved the data collection. Secondary data analysis of the PATH Study Files was approved by the University of California, Los Angeles Institutional Review Board.

### Measures

#### Socio-demographic characteristics

Data on sex (male vs. female) and sexual orientation (lesbian, gay, bisexual or something else vs. heterosexual), was collected during each wave. Sexual orientation was self-identified by asking respondents to answer the following question: “Do you think of yourself as: (a) “Lesbian or gay”, (b) “Straight, that is, not lesbian or gay”, (c) “Bisexual”, or (d) “Something else”. Participants who reported “something else” were probed to provide additional clarifying information (i.e., identifying with other labels such a queer, transgender, in the process of figuring out their sexual orientation, not having a sexuality, not using such labels, or something else). For the purpose of this paper, sexual minorities were defined as lesbian or gay, bisexual or something else, while heterosexuals were defined as straight. Additional demographic data included age, race/ ethnicity, education level, marital status, health insurance status, and annual household income. Age in years was classified as 18–24, 25–34, 35–44, 45–54, and > 55. Race/ethnicity was classified as white non-Hispanic, black non-Hispanic, other non-Hispanic, and Hispanic. Education level was categorized by college or no college. Marital status was categorized as married and non-married. Non-married included widowed, divorced, separated or never married. Annual household income was categorized into income categories: < $25,000, $25,000-49,999, $50,000–99,999 and > $100,000.

#### Hookah and tobacco use patterns and transitions

Ever hookah use was defined as lifetime use. Current hookah use was defined as currently smoking hookah every day or some days (in past 30 days). Study participants were not mutually exclusive to hookah use; that is, some participants who used hookah may also have used other tobacco products including cigarettes, cigar, traditional cigars, filtered cigars, cigarillos, electronic devices, smokeless tobacco (i.e., loose snus, moist snuff, dip, spit, or chewing tobacco), pipe tobacco, snus pouches, or dissolvable tobacco. Categories of single- and multiple-product use for purposes of this paper are described in more detail in the next section.

Among the subset of respondents who reported current hookah only use (no other tobacco products) at Wave 1, four types of transitions to Wave 2 tobacco products were examined: (a) No transition in hookah use (i.e., hookah use at Wave 2 as used at Wave 1); (b) Continued hookah and transitioned to other tobacco product(s) (i.e., hookah plus other tobacco product(s) use at Wave 2); (c) Quit hookah and transitioned to other tobacco product(s) (i.e., no hookah use but other tobacco product(s) use at Wave 2); and (d) Quit all tobacco use (i.e., no use of hookah or any tobacco product at Wave 2).

#### Co-use of tobacco, alternative tobacco products and nicotine delivery systems

To assess for co-use of other tobacco and e-nicotine products, single, dual and poly hookah use were examined using five broad product categories: (a) hookah; (b) cigarettes; (c) e-products (i.e., e-cigarettes, e-hookah (Wave 2 only), e-pipe (Wave 2 only)); (d) smokeless tobacco (i.e., snus, moist snuff, dip, spit, chewing tobacco or dissolvable tobacco); and other combustibles (i.e., traditional cigars, filtered cigars, cigarillos, pipe tobacco). Those who used hookah only were classified as single hookah users. Those who concurrently used hookah plus one other product category were classified as dual hookah users, and those who concurrently used hookah plus 2 or more other product categories were classified as poly hookah users. To emphasize the transition of inclusion of e-products, the following three categories were examined: (a) hookah; (b) hookah plus e-products; and (c) hookah plus other tobacco products, including cigarettes, smokeless tobacco and other combustibles.

### Statistical analyses

Weighted percentages and means along with their corresponding 95% confidence intervals (CI) were calculated using SAS 9.4. Analyses were estimated using the balanced repeated replication (BRR) method with a Fay’s variant to utilize the replicate weights. Comparisons between groups (SM vs. heterosexuals) or between gender subgroups within SM identity groups on demographic variables were determined with Rao-Scott chi-square tests. Supplemental multivariable survey-weighted logistic regression analyses further explored sociodemographic characteristics associated with ever and current hookah use at Wave 1 and Wave 2. Age, gender, sexual orientation, race/ethnicity, education, income, insurance, as well as two-way interactions of sexual orientation with the other predictors, were included in the models.

Additional multivariable survey-weighted multinomial logistic regression models were estimated for selected transition patterns. For these analyses, because of sparse data coverage, age categories were collapsed to 18–24 and 25 years of age or older. Models were developed in a stepwise manner adding one main effect at a time (same predictors as listed for logistic regression), retaining those with *p* < 0.40, and similarly for relevant interactions of sexual orientation with the included effects, until estimation failed. Data would not support estimation of models with all possible transition categories for use status and multi-product use; thus more general categories were defined. The first analysis considered transitions from hookah-only use in Wave 1 to use status in Wave 2, specifically the following patterns: continued use of hookah only, use of other tobacco products in addition to hookah, use of other tobacco products but no hookah use, and no use of any tobacco product. Raw sample size was 322; only main effects of age, gender, and sexual orientation could be included in this model for estimation to be attained. The second transition analysis considered specifically the initiation of electronic product use in Wave 2 for Wave 1 current hookah users. Transition patterns included 1) consistency of product use from Wave 1 to Wave 2 (i.e., continuous hookah-only, continuous hookah plus e-products [with or without other tobacco products], or continuous hookah plus other tobacco products [no e-products]); 2) initiation of e-product use at Wave 2 in addition to continued hookah use; 3) continued hookah use along with any other change in use of other tobacco products; 4) cessation of hookah use. Raw sample size was 743; only the age-by-sexual orientation interaction could be included in the model along with the main effects for age, gender, sexual orientation, race/ethnicity, and health insurance status.

## Results

### Prevalence, socio-demographic and other characteristics

Table 1 presents the prevalence of hookah use by self-reported sexual identity. Overall, SM ever hookah use (Wave 1: 29.3%; Wave 2: 30.9%; weighted) was higher than heterosexual ever hookah use (Wave 1: 16.0%; Wave 2: 15.8%; *p* < 0.05). Similarly, SM current hookah use (Wave 1: 3.6%; Wave 2: 3.2%) was higher than heterosexual current hookah use (Wave 1: 1.3%; Wave 2: 1.0%; p < 0.05). Among SM adults, females reported a higher prevalence of both ever (30.2, 31.5%) and current (4.2, 3.5%) hookah use than males (ever use: 28.0, 32.4%; current use: 2.6, 2.7%) in Wave 1 and Wave 2, respectively. Among heterosexual adults the opposite pattern was observed, with males reporting higher prevalence of ever (19.4, 19.1%) and current (1.7, 1.4%) hookah use than did females (ever use: 12.8, 12.7%; current use: 0.8, 0.7%) for Wave 1 and Wave 2, respectively (*p* < 0.05).

**Table 1:** Prevalence of Hookah Use Stratified by Self-Reported Sexual Identity and Gender

	Ever Use		Current Use	
	Wave 1 Total (***n*** = 10,604)	Wave 2 Total (***n*** = 9021)	Wave 1 Total (***n*** = 1058)	Wave 2 Total (***n*** = 723)
**SM**	1014 (29.31)^a^	901 (30.91)^a^	144 (3.58)^a^	117 (3.15)^a^
**Male**	322 (27.96)^b^	291 (30.37 ^b^	39 (2.6)^b^	33 (2.67)^b^
**Female**	691 (30.23)	609 (31.5)	105 (4.21)	84 (3.5)
**Heterosexual**	9462 (16.01)	8049 (15.79)	910 (1.26)	602 (1.01)
**Male**	5427 (19.44)	4462 (19.12)	593 (1.73)	374 (1.35)
**Female**	4033 (12.75)	3584 (12.66)	317 (0.82)	228 (0.69)

Data represent unweighted numbers (weighted %). Total unweighted numbers for PATH; Wave 1 = 32,548; Wave 2 = 28,362

^a^*P* < 0.0001 comparing weighted rates for SM to heterosexual for ever use (vs. never use) within wave and for current use (vs. no current use) within wave

^b^*P* < 0.0001 for SM comparing weighted rates for males vs. females for ever use (vs. never use) and for current use vs. no current use within wave; and similarly for heterosexuals

Table 2 presents the demographic characteristics of each self-reported sexual identity group in Wave 1 and Wave 2. Both SM and heterosexual participants who used hookah were predominantly aged 18–24 or 25–34 years old. Compared with heterosexual respondents, SM ever and current hookah users were more likely to be female (ever use: in Wave 1 62.9% female vs. 37.0% male, in Wave 2 60.0% vs. 40.0%; current use: in Wave 1 71.8% vs. 28.2%, in Wave 2 65.4% vs. 34.6%). Conversely, the sample of heterosexuals who used hookah was predominantly male (i.e., ever use in Wave 1 59.3% male vs. 40.7% female) (*p* < 0.05).

**Table 2:** Demographic Characteristics of Survey Participants Who Smoke Hookah*

Characteristic	Ever Use				Current Use			
	Sexual Minority Adults		Heterosexual Adults		Sexual Minority Adults		Heterosexual Adults	
	Wave 1	Wave 2	Wave 1	Wave 2	Wave 1	Wave 2	Wave 1	Wave 2
Age								
18–24 Yr	43.11 (39.5–46.72)	42.31 (38.74–45.88)	34.64 (33.37–35.91)	34.36 (32.98–35.73)	67.41 (57.85–76.97)	69.25 (59.04–79.47)	64.89 (60.96–68.82)	63.02 (57.66–68.37)
25–34 Yr	32.71 (29.17–36.24)	35.3 (30.96–39.65)	34.11 (32.76–35.47)	35.69 (34.35–37.03)	24.97 (15.13–34.82)	23.33 (15.36–31.31)	27.75 (24.48–31.02)	29.52 (25.06–33.97)
35–44 Yr	11.56 (9.3–13.82)	11.33 (8.64–14.02)	14.06 (12.94–15.18)	15.05 (13.86–16.25)	4.11 (0.32–7.91)	4.9 (0–10.58)	4.57 (3.05–6.09)	5.12 (3.18–7.07)
45–54 Yr	6.65 (4.64–8.65)	5.88 (3.25–8.5)	8.26 (7.51–9.01)	7.31 (6.55–8.08)	2.14 (0–4.66)	2.51 (0–7.5)	2.00 (0.77–3.22)	1.66 (0.48–2.84)
> 55 Yr	5.98 (3.89–8.07)	5.18 (3.14–7.23)	8.93 (8.2–9.66)	7.58 (6.85–8.32)	1.36 (0–3.95)	0 (0–0)	0.79 (0.12–1.46)	0.68 (0–1.51)
Sex^a^								
Male	37.03 (33.77–40.28)	39.96 (35.76–44.15)	59.31 (58.2–60.42)	58.71 (57.56–59.87)	28.16 (19.05–37.27)	34.56 (25.21–43.91)	66.97 (64.17–69.77)	64.78 (60.79–68.76)
Female	62.97 (59.72–66.23)	60.04 (55.85–64.24)	40.69 (39.58–41.8)	41.29 (40.13–42.44)	71.84 (62.73–80.95)	65.44 (56.09–74.79)	33.03 (30.23–35.83)	35.22 (31.24–39.21)
Race								
White, non-Hispanic	59.67 (55.81–63.53)	55.94 (51.96–59.91)	62.24 (60.67–63.82)	60.73 (59.08–62.38)	58.22 (48.65–67.8)	55.02 (42.85–67.19)	55.47 (51.38–59.57)	48.90 (44.07–53.74)
Black, non-Hispanic	13.47 (10.89–16.05)	12.31 (10.03–14.59)	10.21 (9.27–11.16)	11.31 (10.34–12.29)	11.21 (5.49–16.94)	12.98 (5.7–20.26)	11.17 (8.21–14.13)	12.63 (8.79–16.47)
Other, non-Hispanic	7.7 (5.82–9.57)	7.88 (5.66–10.09)	9.84 (8.75–10.93)	9.98 (9.01–10.96)	6.81 (2.93–10.69)	9.14 (4.62–13.66)	11.19 (8.18–14.19)	15.60 (11.83–19.37)
Hispanic	19.16 (16.16–22.16)	23.88 (20.48–27.28)	17.7 (16.45–18.95)	17.98 (16.67–19.29)	23.75 (14.76–32.73)	22.86 (12.13–33.59)	22.17 (18.8–25.53)	22.86 (18.7–27.02)
Education Level								
No College	32.01 (28.77–35.24)	34.29 (30.33–38.25)	30.21 (28.94–31.48)	30 (28.52–31.47)	36.28 (26.49–46.06)	46.14 (34.05–58.23)	36.3 (33.19–39.41)	37.22 (32.72–41.73)
Some College	67.99 (64.76–71.23)	65.71 (61.75–69.67)	69.79 (68.52–71.06)	70 (68.53–71.48)	63.72 (53.94–73.51)	53.86 (41.77–65.95)	63.7 (60.59–66.81)	62.78 (58.27–67.28)
Marital Status								
Married	–	17.03 (13.97–20.1)	–	31.89 (30.65–33.13)	–	10.57 (4.21–16.93)	–	15.36 (11.58–19.15)
Not Married†	–	82.97 (79.9–86.03)	–	68.11 (66.87–69.35)	–	89.43 (83.07–95.79)	–	84.64 (80.85–88.42)
Annual Household Income								
< $25,000	47.09 (43.49–50.7)	49.27 (45.25–53.3)	35.23 (33.81–36.66)	34.33 (32.79–35.87)	58.96 (48.67–69.26)	54.24 (44.53–63.94)	49.15 (45.7–52.59)	46.16 (41.72–50.6)
$25,000-49,999	24.01 (21.51–26.52)	20.36 (17.08–23.64)	23.03 (22–24.05)	21.91 (20.8–23.02)	24.74 (15.88–33.59)	25.35 (17.61–33.1)	22.8 (19.69–25.92)	18.08 (14.12–22.03)
$50,000-99,000	18.82 (15.79–21.85)	20.36 (17.08–23.64)	23.35 (22.23–24.47)	24.07 (22.78–25.36)	11.96 (4.27–19.64)	12.72 (6.32–19.13)	16.08 (13.24–18.93)	19.7 (16.03–23.37)
> $100,000	10.07 (7.9–12.23)	9.99 (7.57–12.42)	18.39 (17.13–19.65)	19.69 (18.25–21.13)	4.34 (0.69–8)	7.69 (0.69–14.68)	11.97 (9.42–14.51)	16.06 (11.85–20.28)
Health insurance								
No	21.97 (19.24–24.71)	24.08 (20.59–27.57)	18.65 (17.65–19.66)	16.78 (15.55–18.02)	26.48 (19.1–33.86)	29.87 (20.3–39.45)	23.5 (19.53–27.48)	21.87 (17.84–25.91)
Yes	78.03 (75.29–80.76)	75.92 (72.43–79.41)	81.35 (80.34–82.35)	83.22 (81.98–84.45)	73.52 (66.14–80.9)	70.13 (60.55–79.7)	76.5 (72.52–80.47)	78.13 (74.09–82.16)

* African-American adults were oversampled and percentages were weighted to represent the U.S. adult populations. Data are shown as percent (95% CI)

†Including widowed, divorced, separated, or never married

Dash (−) indicates questions were not asked in Wave 1

^a^ *p* < 0.0001 for comparison sexual identity by gender within wave 1 and within wave 2 for those with ever use and for those with current use

In multivariable models, relatively few of the included sociodemographic characteristics (or their interactions with sexual minority status) were consistently statistically significantly associated with ever or current hookah use (Supplemental Table 1). A consistently statistically significant effect across both waves for ever and current use was the gender by sexual minority interaction, with greater likelihood of sexual minority hookah users being female vs. male than among heterosexual hookah users (supporting the simpler comparisons described above). Education was a consistent statistically significant effect, where those with some college were likely to report ever or current use compared to those with no college. In 3 of the 4 models, where the age main effect can be interpreted (Waves 1 and 2 ever use and Wave 1 current use), the older age groups were less likely than the 18–24 year old group to report ever use or current use of hookah, with decreasing likelihood as age increases.

### Hookah smoking transitions from wave 1 to wave 2

**Fig. 1:**
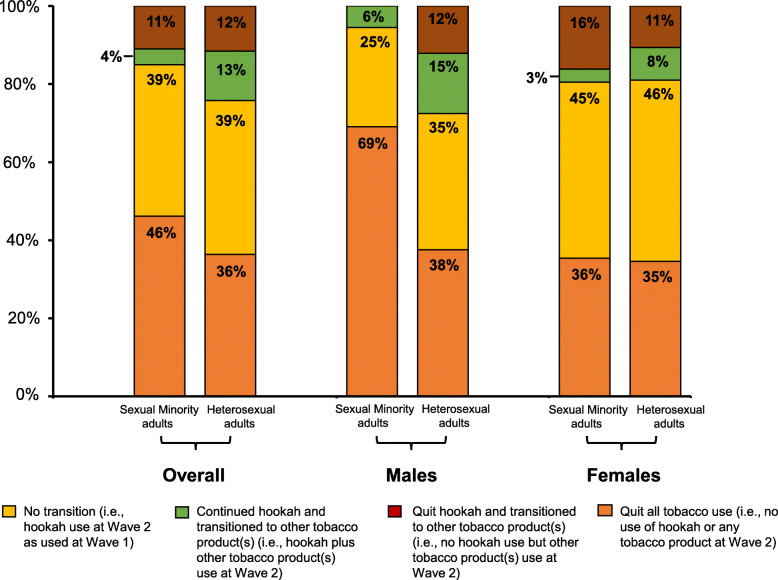
Hookah Smoking Transitions from Wave 1 (2013) to Wave 2 (2014) of the PATH Study. Transitions among current hookah only use reported at Wave 1 were categorized into 4 categories: **a** No transition in hookah use (i.e., hookah use at Wave 2 as used at Wave 1); **b** Continued hookah and transitioned to other tobacco product(s) (i.e., hookah plus other tobacco product(s) use at Wave 2); **c** Quit hookah and transitioned to other tobacco product(s) (i.e., no hookah use but other tobacco product(s) use at Wave 2); and **d** Quit all tobacco use (i.e., no use of hookah or any tobacco product at Wave 2). Estimates were weighted to represent the U.S. adult population

Figure 1 depicts transition patterns by gender and age breakdown among SM adult Wave 1 current hookah-only smokers versus their heterosexual counterparts. Among SM current hookah smokers at Wave 1 who did not use other tobacco products (referred to here as “current hookah-only smokers”), 38.8% continued to smoke only hookah at Wave 2; 4.0% transitioned to use of other tobacco products in addition to hookah use at Wave 2; 11.0% discontinued hookah use and switched to other tobacco products at Wave 2; and 46.2% quit hookah use (and used no other tobacco products) at Wave 2. Among heterosexual adults, the transition pattern was not different from SM adults: among current hookah-only smokers at Wave 1, 39.4% continued to smoke only hookah at Wave 2; 12.7% added other tobacco products use in addition to hookah use in Wave 2; 11.6% discontinued hookah use but used other tobacco products at Wave 2; and 36.4% quit hookah use (and used no other tobacco products) at Wave 2.

Compared with heterosexual men, SM men who reported current hookah use at Wave 1, a greater percentage reported quitting completely at Wave 2 (38% vs. 69%, *p* < 0.05). No significant differences were observed among heterosexual vs. SM women (35% vs. 36%, p = ns). Compared to heterosexual adults, more than half of SM adults age 25–34 years reported quitting hookah use at Wave 2 (43% vs. 63%, *P* < 0.05). Among the younger population (i.e., 18–24 years), 41% of SM adults, compared to 36% heterosexual adults, reported quitting while 47% reported no transition in hookah use at Wave 2, compared to 42% among heterosexuals.

Results of the multinomial model of the four transition categories showed a gender main effect (Supplemental Table 2) with Wave 1 female hookah-only users less likely to discontinue all hookah and tobacco use by Wave 2 or to take up use of other tobacco products; that is, females were more likely to remain consistent in their hookah-only use. Unfortunately, limited data coverage did not allow the inclusion of interaction effects in the multivariable model; thus, the multivariate analysis could not confirm all the comparisons reported in the previous paragraphs.

### Co-use of tobacco, alternative tobacco product and electronic nicotine products

**Fig. 2:**
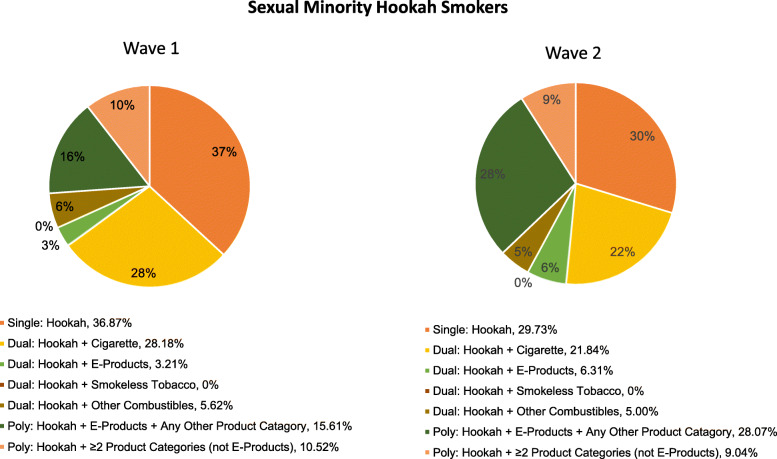
Transitions in Hookah Use and Co-use of Tobacco, Alternative Tobacco Products and E-Products among SM Adult Current Hookah Smokers, arranged according to Single, Dual and Poly Use, Waves 1 and 2 (2013–2014). E-products include e-cigarettes, and e-hookah and e-pipe (Wave 2 only); smokeless tobacco include snus, moist snuff, dip, spit, chewing tobacco or dissolvable tobacco; and other combustibles include traditional cigars, filtered cigars, cigarillos, pipe tobacco. Single use was defined as those who reported hookah only use; dual use was defined as those who concurrently use hookah plus one other product category; and poly use was defined as those who concurrently used hookah plus 2 or more other product categories

**Fig. 3:**
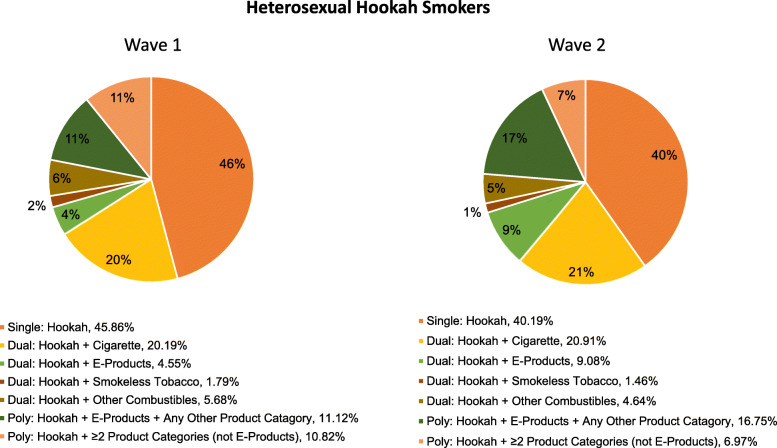
Transitions in Hookah Use and Co-use of Tobacco, Alternative Tobacco Products and E-Products among Heterosexual Adult Current Hookah Smokers, arranged according to Single, Dual and Poly Use, Waves 1 and 2 (2013–2014). E-products include e-cigarettes, e-hookah and e-pipe (Wave 2 only); smokeless tobacco include snus, moist snuff, dip, spit, chewing tobacco or dissolvable tobacco; and other combustibles include traditional cigars, filtered cigars, cigarillos, pipe tobacco. Single use was defined as those who reported hookah only use; dual use was defined as those who concurrently use hookah plus one other product category; and poly use was defined as those who concurrently used hookah plus 2 or more other product categories

**Fig. 4:**
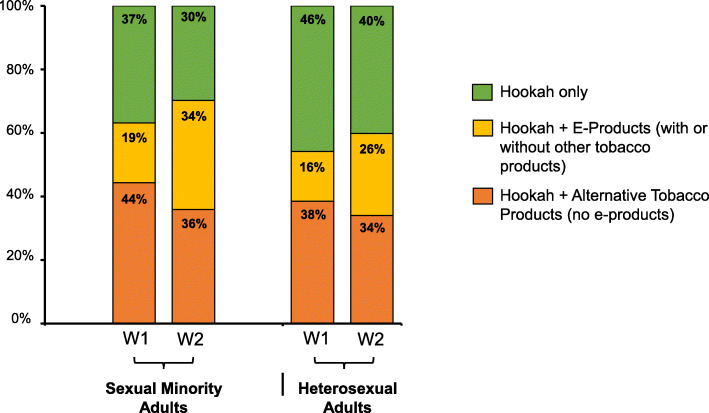
Transitions in Hookah Use and Co-use of E-Products and Alternative Tobacco Products among SM vs. Heterosexual Adult Current Hookah Smokers, Waves 1 and 2 (2013–2014). E-products include e-cigarettes, and e-hookah and e-pipe (Wave 2 only); and alternative tobacco products include cigarettes, smokeless tobacco (i.e., snus, moist snuff, dip, spit, chewing tobacco or dissolvable tobacco) and other combustibles (i.e., traditional cigars, filtered cigars, cigarillos, pipe tobacco)

As shown in Fig. 2 and Fig. 3, cigarettes were the most common tobacco product used in combination with hookah among Wave 1 and Wave 2 SM and heterosexual adult current hookah users. Among Wave 1 SM hookah users, 36.87% reported single hookah use, 37.00% reported hookah dual use and 25.04% poly hookah use. While single and dual hookah use decreased among SM adults in Wave 2 (29.73 and 33.16%, respectively), poly use increased to 35.08%. Among Wave 1 heterosexual adult hookah users, 45.86% reported single hookah use, 32.21% reported hookah dual use and 20.76% poly hookah use. In Wave 2, single hookah use decreased to 40.19% but both dual and poly use increased among heterosexuals in Wave 2 (36.09 and 23.00%, respectively). Dual hookah plus e-products use increased similarly among SM and heterosexual adults from Wave 1 to Wave 2 (increased by 97 and 99%, respectively). A higher percentage of SM adults reported poly hookah use at Wave 2 compared with heterosexual adults (increased by 40% vs. 11%, respectively). While hookah plus e-products use (with or without other tobacco product(s)) in Wave 2 increased significantly among SM adults (increased by 65 and 83%, respectively), both hookah use and hookah plus other tobacco use decreased similarly among SM and heterosexual adults (Fig. 4).

An additional perspective of Wave 2 initiation of multi-product use by Wave 1 current hookah users is provided by the multinomial logistic results of selected transition patterns (Supplemental Table 3); the model included sexual minority status as a predictor, as well as gender, age, race/ethnicity, and health insurance status. Four transition patterns were considered: consistent hookah and other product use across the two waves, initiation of e-products with continued hookah use, other change in multi-product use, and cessation of hookah use. Few differences in transitions were distinguishable in terms of sociodemographic characteristics. In this multivariable model, non-Hispanic Blacks were more likely (OR = 1.22) than were Hispanics to initiate e-products over consistent product use. A significant age by sexual minority status interaction was seen for specifically cessation of hookah use as compared to consistent product use: older sexual minorities were most likely to cease hookah use than maintain a consistent multi-product use pattern (OR = 5.93), calculated from coefficients shown in Supplemental Table 3. This pattern of cessation of hookah use is consistent with the simpler comparative results described in the previous section for the subsample of hookah only users.

## Discussion

Using nationally representative data, we sought to characterize transitions between hookah smoking and use of other tobacco products among SM adults versus their heterosexual counterparts. This study provides two novel insights into these transitions. First, our results demonstrate higher rates of ever and current hookah use among SM adults compared to their heterosexual counterparts. Second, while 46% of SM adults reported quitting hookah smoking at Wave 2, among current hookah users, hookah plus e-product (with or without other tobacco product(s)) use markedly increased at Wave 2 among SM adults (Wave 1: 19%; Wave 2: 34%), compared to increases among heterosexual individuals (Wave 1: 16%; Wave 2: 26%). It is noteworthy that among SM adult current hookah smokers, dual hookah plus e-product use (without other tobacco product(s)) increased by 97% at Wave 2 (Wave 1: 3%; Wave 2: 6%), with comparable trends among heterosexual individuals (Wave 1: 5%; Wave 2: 9%).

While the investigation into the cause of the recent epidemic of vaping-induced deaths and illness is still ongoing [21, 22], our findings highlight vital trends regarding the rapid uptake of vaping—using various e-products such as e-cigarettes, e-hookahs and e-pipes—among SM hookah smokers. In a two-year period, our nationally representative findings show that hookah plus e-product use (with or without other tobacco product(s)) increased by 83% among SM adults, compared to 65% among heterosexual individuals. In light of these findings, and because there is limited evidence for interventions to address common misperceptions on potential hookah harms [23, 24], our study emphasize the need for strong efforts to increase awareness of the harmful effects of hookah as well as vaping, targeted towards sexual minority populations. Our findings also illustrate the importance of feasible and effective health education programing and communication efforts, specifically tailored to SM communities. For example, special programing that could potentially prevent the onset or continued use of hookah and vaping products and assist with cessation programs aimed at reaching SM populations. Indeed, evidence suggests that few anti-tobacco campaigns have been designed specifically to reach sexual minority populations [25].

Use of alternative tobacco products such as hookah has risen abruptly in the past decade [9, 26]. Few studies have examined hookah use among SM populations. Prior analysis of PATH data from Wave 1 found that SM individuals had higher odds of hookah use compared to heterosexual individuals [10]. Similarly, nationally representative data from Legacy’s Young Adult Cohort Study show that ever hookah use was significantly higher among SM respondents compared with those who identified as heterosexuals [27]. Our analyses confirm these findings by showing that over a two-year period, SM adults continue to have significantly higher rates of hookah use compared with heterosexual adults. Furthermore, our analysis extends these findings by demonstrating that a larger percentage of SM adult, specifically male hookah smokers, compared to heterosexuals, reported quitting hookah smoking at Wave 2. While is it unknown whether these individuals may return to use hookah, future analysis of additional waves of the PATH study may provide further insight into longer-term patterns of hookah use within SM populations.

There is growing concern that hookah smoking may function as a gateway to other tobacco products and harmful substances. Recent prospective analysis from the PATH study 2013–2015 indicate that hookah use is independently associated with subsequent smoking in the year ahead [28]. This finding is consistent with other studies that demonstrate hookah use is associated with more than double the odds of subsequent initiation of cigarette smoking [29]. Our analyses demonstrate that a large majority of SM current hookah smokers (63% in Wave 1 and 70% in Wave 2) reported using hookah plus other tobacco products, with cigarettes being the most common tobacco product used in combination with hookah. While multiple factors may explain our findings, flavored tobacco products have been previously demonstrated to serve as starter products to regular tobacco use [30]. Indeed, sexual minority status has been shown to be associated with use of flavored tobacco products [31, 32], and evidence show that the tobacco industry has selectively targeted the marketing of products to sexual minority individuals [12, 13, 15, 17]. In addition to tobacco and menthol flavors, hookah tobacco come in fruit, candy, and alcohol flavors and while the 2009 Family Smoking Prevention and Tobacco Control Act banned characterizing flavors other than menthol in cigarettes, this ban does not extend to hookah [33]. Our findings build upon previous work highlighting the need for robust regulation to reduce flavored tobacco appeal specifically among SM communities.

There are several limitations to this study. Respondents’ smoking status was not biochemically verified. Although this study focused exclusively on SM adults, it is important to note gender differences within SM and heterosexual samples when addressing transitions. Combining sexual minority subgroups (i.e., lesbian women, bisexual men) may mask unique differences with regards to hookah use prevalence and transitions, and may obscure subgroup specific health needs. Because the PATH questionnaire’s ‘something else’ category encompasses a highly heterogeneous group that may not necessarily represent the definition of “sexual minority” (i.e., genderqueer people), future research is needed to include specific questions to identify gender diverse individuals and better understand hookah tobacco trends among gender minorities as well as sexual minorities [34]. PATH Study data were self-reported and therefore responses may underrepresent the SM community because of the related-stigma surrounding sexual orientation. Further exploration is needed with longitudinal models that can accommodate the complex survey weights as well as capture behavior change and time-dependent covariates.

## Conclusions

This study is one of the first to characterize quitting as well as transitions between hookah smoking and use of other tobacco products among SM adult hookah smokers using a nationally representative sample in the United States. In addition to higher rates of hookah use among SM adults, higher percentages of SM adults transitioned to hookah plus e-product use between 2013 and 2015 compared to their non-minority peers. Considering our findings in light of the study limitations and the context of the limited literature, future work should aim to further examine mechanisms that drive the higher rates of hookah use among SM individuals and how these drivers may differ by unique SM subgroups (i.e., socialization/affiliation versus stress processes may differ by subgroup). Finally, information regarding the harmful effects of hookah use should be tailored to reach diverse sexual minority communities.

## Supplementary Information

**Additional file 1: Supplemental Table 1**. Sociodemographic Predictors of Ever and Current Hookah Use Waves 1 and 2 (2013-2014). **Supplemental Table 2**. Sociodemographic Predictors for Wave 1 Hookah-Only Users Transition Patterns. **Supplemental Table 3**. Sociodemographic Predictors of Transition Patterns Wave 1 to Wave 2 (Including Uptake of E-Products)

## Data Availability

The datasets generated and/or analysed during the current study are available from the Population Assessment of Tobacco and Health Study Public-Use Files, https://www.icpsr.umich.edu/icpsrweb/NAHDAP/studies/36498

## Abbreviations

PATH: Population Assessment of Tobacco and Health
SM: Sexual minority
