# Supplementary material for: Transitions in hookah (Waterpipe) smoking by U.S. sexual minority adults between 2013 and 2015: the population assessment of tobacco and health study wave 1 and wave 2
Source: BMC Public Health. 2021 Mar 5;21:445. doi: 10.1186/s12889-021-10389-5 (PMC7934533; doi:10.1186/s12889-021-10389-5)
Supplement: Supplementary file 1 — Additional file 1: Supplemental Table 1. Sociodemographic Predictors of Ever and Current Hookah Use Waves 1 and 2 (2013-2014). Supplemental Table 2. Sociodemographic Predictors for Wave 1 Hookah-Only Users Transition Patterns. Supplemental Table 3. Sociodemographic Predictors of Transition Patterns Wave 1 to Wave 2 (Including Uptake of E-Products) [file 12889_2021_10389_MOESM1_ESM.docx]

**Supplemental Table 1.** Sociodemographic Predictors of Ever and Current Hookah Use Waves 1 and 2 (2013-2014)

| **Parameter** | | **Ever Use** | | | | **Current Use** | | | |
| --- | --- | --- | --- | --- | --- | --- | --- | --- | --- |
|  |  | **Wave 1** | | **Wave 2** | | **Wave 1** | | **Wave 2** | |
|  |  | **Coeff (SE)** | **p-value** | **Coeff (SE)** | **p-value** | **Coeff (SE)** | **p-value** | **Coeff (SE)** | **p-value** |
| Intercept |  | -0.24 (0.07) | 0.001 | -0.18 (0.08) | 0.026 | -2.40 (0.12) | <0.001 | -2.76 (0.13) | <0.001 |
| Gender (ref: male) | Female | -0.53 (0.04) | <0.001 | -0.50 (0.04) | <0.001 | -0.74 (0.08) | <0.001 | -0.54 (0.10) | <0.001 |
| Age (ref: 18-24) | 25 - 34 | -0.59 (0.05) | <0.001 | -0.55 (0.05) | <0.001 | -1.18 (0.10) | <0.001 | -1.11 (0.13) | <0.001 |
|  | 35 - 44 | -1.68 (0.07) | <0.001 | -1.61 (0.07) | <0.001 | -2.86 (0.19) | <0.001 | -2.79 (0.23) | <0.001 |
|  | 45 - 54 | -2.31 (0.07) | <0.001 | -2.41 (0.08) | <0.001 | -3.80 (0.35) | <0.001 | -3.87 (0.43) | <0.001 |
|  | ≥55 | -2.89 (0.06) | <0.001 | -3.12 (0.07) | <0.001 | -5.21 (0.53) | <0.001 | -5.41 (0.77) | <0.001 |
| Race  (ref: non-Hisp.white) | Hispanic | 0.08 (0.07) | 0.241 | 0.08 (0.07) | 0.290 | 0.11 (0.11) | 0.308 | 0.29 (0.14) | 0.041 |
|  | Non-Hisp. Black | -0.20 (0.07) | 0.008 | -0.08 (0.08) | 0.263 | -0.12 (0.17) | 0.476 | 0.18 (0.20) | 0.382 |
|  | Non-Hisp. other | 0.07 (0.08) | 0.366 | -0.010 (0.09) | 0.918 | 0.15 (0.15) | 0.320 | 0.60 (0.16) | <0.001 |
| Sexual identity  (ref: heterosexual) | Sexual minority | 0.13 (0.20) | 0.527 | 0.29 (0.24) | 0.223 | -0.07 (0.40) | 0.858 | 0.71 (0.38) | 0.065 |
| Education  (ref: no college) | Some college | 0.60 (0.05) | <0.001 | 0.54 (0.06) | <0.001 | 0.36 (0.08) | <0.001 | 0.24 (0.12) | 0.043 |
| Health insurance  (ref: no) | Yes | -0.10 (0.05) | 0.040 | -0.05 (0.07) | 0.446 | -0.08 (0.13) | 0.541 | -0.07 (0.13) | 0.587 |
| Household income  (ref: <$25,000) | ≥$100,000 or more | 0.07 (0.07) | 0.310 | -0.00 (0.07) | 0.952 | -0.33 (0.14) | 0.020 | -0.13 (0.17) | 0.457 |
|  | $50,000-99,999 | 0.01 (0.05) | 0.823 | -0.12 (0.05) | 0.029 | -0.14 (0.10) | 0.170 | -0.37 (0.14) | 0.008 |
|  | $25,000-49,999 | -0.09 (0.06) | 0.111 | -0.17 (0.06) | 0.007 | -0.46 (0.12) | <0.001 | -0.27 (0.14) | 0.042 |
| Gender*sexual identity | Female/sexual minority | 0.48 (0.13) | 0.002 | 0.34 (0.17) | 0.047 | 1.11 (0.26) | <0.001 | 0.65 (0.26) | 0.013 |
| Education*sexual identity | Some college/sexual minority | 0.20 (0.15) | 0.175 | 0.21 (0.21) | 0.322 | 0.17 (0.26) | 0.520 | -0.19 (0.34) | 0.570 |
| Age*sexual identity | 25-34/sexual minority | -0.21 (0.15) | 0.148 | -0.04 (0.17) | 0.825 | -0.08 (0.33) | 0.816 | -0.25 (0.30) | 0.409 |
|  | 35-44/sexual minority | 0.07 (0.18) | 0.712 | -0.04 (0.20) | 0.842 | 0.36 (0.55) | 0.511 | 0.34 (0.82) | 0.681 |
|  | 45-54/sexual minority | -0.03 (0.24) | 0.889 | 0.18 (0.29) | 0.532 | 0.90 (0.86) | 0.300 | -11.24 (4.47) | 0.012 |
|  | ≥55/sexual minority | 0.28 (0.29) | 0.320 | 0.55 (0.28) | 0.046 | 1.74 (1.36) | 0.189 | -9.46 (4.34) | 0.030 |
| Race*sexual identity | Hispanic/sexual minority | -0.26 (0.18) | 0.155 | -0.11 (0.18) | 0.543 | -0.02 (0.32) | 0.953 | -0.65 (0.40) | 0.108 |
|  | Non-Hispanic B/lack/sexual minority | 0.21 (0.24) | 0.374 | 0.26 (0.20) | 0.205 | -0.08 (0.38) | 0.824 | -0.15 (0.40) | 0.715 |
|  | Non-Hispanic other/sexual minority | -0.66 (0.32) | 0.039 | -0.16 (0.30) | 0.589 | -0.61 (0.47) | 0.193 | -0.54 (0.42) | 0.196 |
| Insurance*sexual identity | Yes/sexual minority | 0.06 (0.14) | 0.677 | -0.18 (0.16) | 0.264 | -0.17 (0.21) | 0.422 | -0.28 (0.30) | 0.355 |
| Income*sexual identity | ≥$100,000/sexual minority | 0.11 (0.23) | 0.635 | -0.19 (0.30) | 0.532 | -0.33 (0.57) | 0.570 | -0.04 (0.60) | 0.943 |
|  | 50,000-99,999/sexual minority | 0.11 (0.14) | 0.439 | -0.02 (0.17) | 0.930 | 0.25 (0.32) | 0.426 | 0.65 (0.33) | 0.050 |
|  | $25,000-49,000/sexual minority | 0.27 (0.21) | 0.207 | 0.26 (0.22) | 0.233 | 0.22 (0.44) | 0.619 | 0.15 (0.39) | 0.696 |

Wald chi square (df=27)=4271.09, p<0.001 [Wave 1 Ever]; 3482.28, p<0.001 [Wave 2 Ever]; 1092.91, p<0.001 [Wave 1 Current]; 536.47, p<0.001 [Wave 2 Current].

**Supplemental Table 2.** Sociodemographic Predictors for Wave 1 Hookah-Only Users Transition Patterns

| **Parameter** |  | **Model:**  **Transition from Wave 1 Hookah-only to wave 2 category (ref: wave 2 Hookah-only use [H-only]** | **Coeff (SE)** | **p-value** | **Odds Ratio** |
| --- | --- | --- | --- | --- | --- |
| Intercept |  | No Tobacco use of any kind [No T, no H] | 0.02 (0.19) | 0.931 | 1.02 |
| Intercept |  | No Hookah use + use of other tobacco products [T, no H] | -1.48 (0.47) | 0.002 | 0.23 |
| Intercept |  | Hookah + use of other tobacco product [H+T] | -0.77 (0.28) | 0.007 | 0.47 |
| Gender  (ref: male) | Female | No H, no T | -0.62 (0.31) | 0.046 | 0.54 |
|  | Female | T, no H | -0.20 (0.51) | 0.697 | 0.82 |
|  | Female | H+T | -1.12 (0.46) | 0.015 | 0.33 |
| Age category (ref: 18-24) | >=25 | No H, no T | 0.342 (0.30) | 0.258 | 1.41 |
|  | >=25 | T, no H | 0.96 (0.53) | 0.067 | 2.62 |
|  | >=25 | H+T | -0.10 (0.53) | 0.853 | 0.91 |
| Sexual identity (ref: heterosexual) | Sexual minority | No H, no T | 0.64 (0.44) | 0.140 | 1.90 |
|  | Sexual minority | T, no H | -0.99 (1.31) | 0.453 | 0.37 |
|  | Sexual Minority | H+T | -0.16 (0.81) | 0.846 | 0.86 |

Wald chi square (df=18)=29.720, p=0.040

Transition categories, from Wave 1 Hookah-only to: wave 2 Hookah-only [H-only, reference category], Hookah+one or more of any other tobacco product [H+T], one or more other tobacco product but no Hookah [T, no H], no hookah and no other tobacco product [No H, no T].

**Supplemental Table 3.** Sociodemographic Predictors of Transition Patterns Wave 1 to Wave 2 (Including Uptake of E-Products)

| **Predictor** |  | **Transition**  **(ref: no change in product use in wave 2 [No chg])** | **Coeff(SE)** | **p-value** | **Odds Ratio** |
| --- | --- | --- | --- | --- | --- |
| Intercept |  | Stopped hookah use (No H) | 0.26 (0.25) | 0.302 | 1.27 |
| Intercept |  | Any other change, but continue hookah use [H-other] | -1.32 (.46) | 0.004 | 0.58 |
| Intercept |  | Uptake of e-products, continue hookah [H+uptakeE] | -1.26 (0.49) | 0.010 | 0.62 |
| Gender  (ref: male) | Female | No H | -0.23 (0.20) | 0.241 | 0.85 |
|  | Female | H-other | 0.03 (0.42) | 0.937 | 0.52 |
|  | Female | H+uptakeE | -0.49 (0.36) | 0.182 | 1.22 |
| Age category  (ref: 18-24) | >=25 | No H | 0.32 (0.19) | 0.085 | 0.61 |
|  | >=25 | H-other | -0.22 (0.45) | 0.641 | 0.81 |
|  | >=25 | H+uptakeE | -0.41 (0.45) | 0.357 | 2.70 |
| Sexual identity (ref: heterosexual) | Sexual minority | No H | -0.61 (0.32) | 0.053 | 0.45 |
|  | Sexual minority | H-other | -0.80 (0.82) | 0.332 | 0.90 |
|  | Sexual minority | H+uptakeE | -0.22 (0.62) | 0.724 | 1.84 |
| Health insurance  (ref: no) | Yes | No H | 0.24 (0.23) | 0.296 | 6.02 |
|  | Yes | H-other | -0.54 (0.42) | 0.200 | 2.86 |
|  | Yes | H+uptakeE | -0.48 (0.43) | 0.269 | 2.98 |
| Race/ethnicity (ref: non-Hispanic white) | Hispanic | No H | -0.16 (0.21) | 0.442 | 1.27 |
|  | Hispanic | H-other | -0.66 (0.51) | 0.202 | 0.58 |
|  | Hispanic | H+uptakeE | 0.20 (0.47) | 0.669 | 0.62 |
|  | Non-Hisp. Black | No H | -0.49 (0.31) | 0.118 | 0.85 |
|  | Non-Hisp. Black | H-other | -0.21 (0.68) | 0.761 | 0.52 |
|  | Non-Hisp. Black | H+uptakeE | 0.99 (0.46) | 0.030 | 1.22 |
|  | Non-Hisp. other | No H | -0.80 (0.31) | 0.010 | 0.61 |
|  | Non-Hisp. other | H-other | -0.10 (0.62) | 0.871 | 0.81 |
|  | Non-Hisp. other | H+uptakeE | 0.61 (0.60) | 0.310 | 2.70 |
| Interaction—  Age x sexual orientation (ref: younger/ heterosexual) |  | No H | 1.79 (0.66) | 0.007 | 0.45 |
|  |  | H-other | 1.05 (1.47) | 0.475 | 0.90 |
|  |  | H+uptakeE | 1.09 (1.16) | 0.347 | 1.84 |

Wald chi square (df=24)=60.393, p=<.001

Product categories considered: hookah-only, hookah + electronic tobacco products (with or without other tobacco products), hookah + other non-electronic tobacco product (no electronic products), no hookah use at wave 2 (with or without other electronic or non-electronic tobacco products)

Transition categories: no change in use of product categories wave 1 to wave 2 [No chg]; upake of e-products at wave 2 with continued use of hookah (with or without other non-electronic products) [H+uptakeE]; any other change with continued hookah use [H-other]; stopped hookah at wave 2 (with or without e-product or other tobacco product use at wave 2) [No H]
